# How body concerns, body mass, self-rated health and self-esteem are mutually impacted in early adolescence: a longitudinal cohort study

**DOI:** 10.1186/s12889-021-10553-x

**Published:** 2021-03-12

**Authors:** Eivind Meland, Hans Johan Breidablik, Frode Thuen, Gro Beate Samdal

**Affiliations:** 1 Department of Global Public Health and Primary Care, Research Group for General Practice, Årstadveien 17, 5018 Bergen, Norway; 2 Department of Research and Development, District General Hospital of Førde, 6800 Førde, Norway; 3 Center for Evidence-Based Practice, Western Norway University of Science, Inndalsveien 28, 5063 Bergen, Norway; 4 Faculty of Health, VID Specialized University, 5145 Fyllingsdalen, Bergen, Norway

**Keywords:** Adolescence, Body dissatisfaction, Body weight concern, Body mass index, Self-esteem, Self-rated health

## Abstract

**Background:**

Impaired self-rated health (SRH) and self-esteem (SE) in adolescents are associated with increased body mass index (BMI). These associations are often studied using cross-sectional designs; we performed a longitudinal cohort survey to examine them.

**Methods:**

A longitudinal cohort study of 1225 Norwegian high school students, with SRH, SE and BMI as primary outcomes. We reported the results from temporal causal and residual change analyses separately, with odds ratios (ORs) and standardised regression coefficients (b) and 95% confidence limits.

**Results:**

Body and weight concerns had unfavourable effects on SRH and SE, which both had favourable effects on each other. Increased BMI had unfavourable effects on SRH, but less so on SE. Body and weight concerns impacted SE change only among girls. Paradoxically, the intention of becoming thinner was associated with an increase in BMI, and the intention of becoming fatter predicted a decrease in BMI during the 2 years. SE and SRH were associated with a leaner body after 2 years.

**Conclusions:**

This study confirms that body concerns had unfavourable effects on subjective health, and that positive self-concepts predicted a leaner body. Health promotion strategies built on body acceptance should be increasingly emphasised in clinical and public health practice.

**Supplementary Information:**

The online version contains supplementary material available at 10.1186/s12889-021-10553-x.

## Background

According to most researchers in the field, being overweight or obese during childhood and adolescence is associated with non-communicable diseases and even with mortality [1]. Some researchers maintain, however, that the causation is complex and insufficiently understood [2]. Weight-related factors like socio-economic deprivation, chronic stress, physical inactivity, body concern and stigmatisation may mediate the associations between weight and morbidity and mortality [2, 3].

The complex associations between these factors have been studied in different populations. Body dissatisfaction (concerns about weight and appearance) among college students is associated with low psychosocial functioning manifest as, for example, eating disturbances, poor self-esteem (SE) and social discomfort [4]. Weight stigmatisation is related to increased stress, negative body image and reduced SE, binge eating, decreased physical activity, weight gain, and increased morbidity among adults [5] and with weight gain among adolescents [6]. Young populations may be especially vulnerable to the side-effects of a focus on weight, as body dissatisfaction and subjective health impairments (evaluation of one’s own health status) are strongly interrelated and prevalent within these groups [7]. Appearance concerns are important for SE (belief and confidence in one’s own ability and value) especially among girls [8], and adolescents may be vulnerable to the stigmatising side-effects of well-intentioned public health communication on weight [9].

In spite of this, dieting and weight loss recommendations are popular and prevalent in many public health policies, including the Norwegian Directorate of Health’s guideline for treating excess weight and obesity [2, 10].

Some researchers maintain that obesity can be understood as an eating disorder with similar causal factors as anorexia nervosa and binge eating. Improving body acceptance and self-confidence may therefore be important health promotive elements in preventive efforts to combat obesity [11, 12].

Short-term weight loss interventions may improve health, but the improvements may just as easily be attributed to other factors, such as physical activity and healthy eating [2]. The fact that health benefits from weight loss rarely show a dose-response relationship may indicate that it is the behaviour change and not the weight loss that provides the effects. Long-term studies show complete weight regain in most participants, resulting in compromised physical and psychological health associated with weight cycling [13].

The claimed causal effects between weight loss and morbidity and mortality were extensively studied in the Look Ahead trial, involving more than 5000 overweight and obese participants with type 2 diabetes. The intervention group decreased their body weight compared with controls, and showed improved diabetes-related metabolic factors. However, no significant effects were detected concerning cardiovascular morbidity and mortality in this adult and elderly population at high risk for such disease [14].

In younger age groups, where disease prevalence is low, self-rated health (SRH) is an important indicator for health. It is a precursor for impaired health later in life even in younger populations [15, 16]. Likewise, SE is an important resilience factor in adolescence associated with emotional wellbeing, whereas deteriorating SE predicts psychological morbidity, e.g. depression and anxiety, later in life [17]. Both these measures are sex dependent as girls are more likely to report impaired SRH and SE compared with boys.

The health consequences of body concerns have mainly been studied among females, and often among groups with eating disorders. The present survey enabled us to examine how body concerns, SE, SRH and body mass index (BMI) were interrelated in a general adolescent population during 2 years. We were also able to adjust for socioeconomic status (SES), acknowledging that this measure has a consistent association with subjective health [18].

Specifically, we set out to examine:

- How body weight and body shape concerns predicted SRH and SE in a general adolescent population after 2 years;
- How the reciprocal associations between these outcomes were during the 2 years; and
- How predictors influenced *change* in the outcomes during the two-year time span.

## Methods

### Participants

**Fig. 1:**
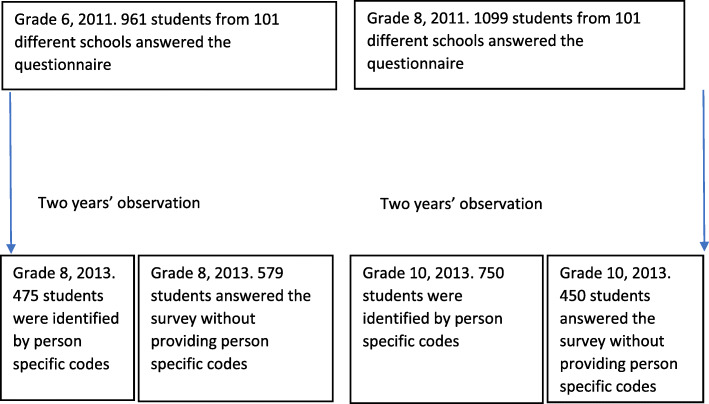
Students participating in the longitudinal cohort study from elementary and junior high schools in the former county of Sogn og Fjordane from 2011 to 2013. The longitudinal study was part of two cross sectional studies in 2011 and 2013. Therefore, the total number of participants differs between the two years

We invited all municipalities in the former county of Sogn og Fjordane in western Norway to participate in the survey, and all except one accepted the invitation. In 2011, 67% of 3075 students in grade 6 and grade 8 (2060 students) took part. In 2013, 72% of 4538 students from grades 6, 8 and 10 responded (2254 students from grades 8 and 10), and 101 different schools participated in both surveys. The study design is outlined in Fig. 1, and is described in detail in a former study [19].

The main reason for non-participation was absence from school on the day of data collection. The participation across grade levels was broadly similar: 1001 students in grade 6; 1054 grade 8; and 1200 in grade 10. The students in grades 8 and 10 in 2013 answered the same survey in 2011, but we only identified 1225 of them by person-specific codes across the two time points due to insufficient coding. The coding insufficiencies were more prevalent among the youngest cohort (11 years old in 2011). We followed a total of 612 boys and 613 girls; 475 from 6th to 8th grade and 750 from 8th to 10th grade, i.e. 1225 students with an almost identical sex distribution across the two cohorts. The proportion identified in the 6th to 8th grade cohort was 45% (475/1054, 95% CI: 42–48), whereas in the oldest cohort, 63% were identified (750/1200, 95% CI: 60–65) (See Fig. 1 and Table 1). The former county of Sogn og Fjordane has a very homogenous population. Only 109 of the students had one parent born abroad, and only 38 had parents who were both born outside Norway. The vast majority of them came from European countries.

**Table 1:** Sex, age, socioeconomic status (family affluence), self-rated health, self-esteem, BMI, and body concerns among 1225 students from Sogn og Fjordane surveyed at two time-points (2011 and 2013

Variables	N (%) or valid responses	Response options (Likert scale)	Mean (SD)	Min - max	Cronbach’s alpha
Adjusting variables					
***Sex***					
Girls	613 (50)				
Boys	612 (50				
***Grade level***					
6. grade in 2011 (11 years of age)	475 (39)				
8. grade in 2011 (13 years of age)	750 (41)				
***Self-rated family affluence, 2011***	1181	5 (1–5)	2.2 (0.7)	1–5	
Outcomes					
***Self rated health, 2013***					
Very good	349 (30)				
Good	597 (51)				
Not so good and bad	231 (19)				
***Self rated health, 2011***					
Very good	412 (34)				
Good	677 (56)				
Not so good and bad	117 (10)				
***Self-esteem, 2013***	1207	4 (1–4)	3.0 (0.6)	1.0–4.0	0.90
***Self-esteem, 2011***	1191	4 (1–4)	3.1 (0.5)	1.2–4.0	0.86
***BMI, 2013***	1037		20.6 (4.2)		
***LogBMI, 2013***	1037		1.3 (0.08)	1.1–1.8	
***LogBMI, 2011***	941		1.3 (0.07)	1.0–1.8	
Predictor variables					
***Want to change something about body***					
Yes	625 (52)				
No	580 (48)				
***Evaluate my body***					
It is OK or I don’t think about it	734 (62)				
It is too thin	137 (12)				
It is too fat	314 (27)				
***Dieting***					
No, my weight is fine	747 (62)				
No, I need to gain weight	82 (7)				
No, but I need to lose weight	144 (12)				
Yes. I am dieting	225 (18)				

### Procedures

Self-report questionnaires were distributed by teachers during class hours. The youngest age group (6th grade) used two class hours to fill in the questionnaire, whereas the oldest (8th and 10th grade) used 1 h. Teachers were available to clarify possible misunderstandings. The questionnaires were delivered sealed in blank envelopes. Two hundred and eighty three variables originating from seventy three questions were included.

This cohort of 1225 students, surveyed and identified at both time-points, comprised 49% of the original group of students measured in grade 6, and 68% of the students measured in grade 8 in 2011. Most students completed the questionnaires. Students reported their weight and height in the questionnaire, and these measures were somewhat insufficiently reported. BMI computation was only possible for 941 participants in 2011 and 1037 in 2013.

### Measures

Most of the questions in the survey were from the international World Health Organisation (WHO) led project Health in School-Aged Children (HBSC), and they proved valid and reliable in previous studies from Norway [7, 20]. We have provided an English version of the part of the questionnaire used in the present study in Additional file 1.

SRH is a one-question item pertaining to how the individual estimates their current health status. Participants answered the question on a four-point Likert scale from ‘very good’ to ‘bad’. Only 12 participants reported bad health. Therefore, we merged this category with the category ‘not so good’ forming a three-level ordinal variable with increasing values indicating improved health. This one-item question has proved valid in many studies, including in Norwegian settings [7, 15, 16].

SE was measured with Rosenberg’s self-esteem scale [21]. The scale had 10 questions designed to measure the students’ overall evaluation of their worthiness as human beings (e.g. ‘on the whole, I am satisfied with myself’). The answers ranged from ‘strongly disagree’ to ‘strongly agree’ on a four-point Likert scale. We recoded the answers to give the same direction for all answers, indicating increased values for improved SE. The Cronbach’s alpha of the SE measure in 2013 was 0.90 as Table I shows. The questions pertaining to body and weight concerns were well understood and answered satisfactorily, as can be seen in Table 1.

Socioeconomic status (SES) was measured with one question regarding family finances. The pupils were asked about how ‘well off’ they considered their family to be. The answer alternatives varied from ‘very well off’ to ‘badly off’ on a 5-point Likert scale. Increasing values indicated less affluence. This question has been used to measure SES among adolescents and has been associated with subjective health complaints in several studies [18].

Body weight concern was tapped by two questions. The first question pertained to how participants evaluated their body with the alternatives: ‘It is OK’, ‘I don’t think about it’, ‘It is too thin’, and ‘It is too fat’. The two first alternatives were combined yielding three alternatives. The second question pertained to dieting. The alternatives were ‘No, my weight is fine’, ‘No, I need to gain weight’, ‘No, but I need to lose weight’, and ‘Yes, I am dieting’. Body shape concern was tapped with one ‘Yes/No’ question asking if there was something about their body the students wanted to change. These questions originate from the WHO-led HBSC study, and have also proved reliable and valid in Norwegian settings [19, 20]. The questions originate from items used in research about eating disorders [22].

An earlier study of the same population showed that less than 5% of the variance in the variables was accounted for at the level of the school class to which the students belonged [19]. Therefore, we abstained from performing multi-level analyses.

### Analysis

We presented the population with frequencies and we calculated the mean scores for the SE scale variable. To be included in the mean score calculation, respondents were required to answer at least 50% of the items constituting each variable. One-item variables and construct variables with missing values were excluded from the analyses. The variables were normally distributed, except for BMI (skewness 2.8 in 2013). Therefore, we log-transformed this variable when we applied it as an outcome measure. The log-transformed variable was normally distributed, as was the SE mean score. The variable tapping self-rated family affluence had a normal distribution with skewness − 0.6. We entered this variable in the regression models as a continuous variable.

We performed ordinal logistic analyses with the three level SRH as outcome. For the SE and LogBMI outcomes we performed linear regression analyses. To judge how the adjusting variables influenced the outcomes, we first entered them in the models all together, and noted the explained variance for the adjusting variables together. Thereafter, we entered the predictors one by one into the model, and reported the explained variance in the tables. Thereby, we were able to judge the added explained variance and the impact of each predictor. In the residual change analyses, we entered the outcome variable as it was reported in 2011 to judge if the predictors also impacted the *change* in outcomes after 2 years. Also, for these analyses, we reported the explained variance as noted above. In the ordinal logistic regression analyses we used Nagelkerke pseudo-explained variance, well aware that this variable cannot be interpreted as straightforwardly as R^2^ from linear regression analyses.

When categorical variables were used in linear regression analyses, we recoded them into dummies if they contained three or more levels. The Chi^2^ values from cross table analyses between the body shape and body weight concern variables were all > 250. To avoid multi-collinearity, we abstained from performing multi-variable analyses with these variables simultaneously in the models. All the other variables were correlated with Spearman’s rho < 0.35, ruling out multi-collinearity for these measures.

In stratified analyses for each sex, we found that the associations were stronger for girls than boys in general, especially for SE. The confidence intervals (CIs) were overlapping for most of the associations, although some of the CIs did not overlap for the associations between body shape and weight concern and SE. Therefore, we performed separate analyses for boys and girls for the predictor analyses with SE as outcome. In the analyses with SRH and BMI as outcomes we introduced sex as an adjusting variable and performed the analyses with both sexes together.

In the linear regression analyses, with SE and LogBMI as outcomes, we studied the residuals for deviation from normality. We tested the ordinal logistic models, with SRH as outcome, for parallel lines. Residuals were normally distributed for SE, whereas slight deviations were revealed for LogBMI. Tests for parallel lines were all insignificant. We maintain that small deviations from normality are not problematic in regression models aimed to model mean values of the outcome as a function of the covariates. Our objective was not to predict individual outcomes based on the covariates. Therefore, we claim that the assumptions of linear regression and ordinal logistic regression were satisfied.

We used IBM SPSS 25 for the analyses. A *p*-value < 0.05 was accepted as significant.

## Results

We compared the cohort participants in the present study with all study participants in the 2013 study from grades 8 and 10 (see Fig. 1) in order to reveal any systematic drop-out. SES in this cohort was similar to the SES of all pupils surveyed in 2013. Body dissatisfaction and subjective health are increasingly impaired during early adolescence [7]. Therefore, the greater drop-out rate in the younger cohort led to an overestimated prevalence of impaired subjective health and body concerns in this cohort, compared with the total population surveyed in 2013.

Table 2 demonstrates that BMI and SE in 2011 impacted later SRH strongly and the impact was statistically significant (OR = 0.9, 95% CI = 0.9,0.9 and OR = 2.5, CI = 2.0,3.2 respectively). The weight and body concern variables had an almost equally strong impact on SRH 2 years later. The table reveals that being content with the body predicted improved SRH with ORs from 1.8 (CI = 1.4,2.3) to 2.3 (CI = 1.7,3.1). It is worth noting that the self-evaluation of being too thin and needing to gain weight was not associated with impaired SRH, with ORs similar to those associated with being content. The stratified analyses did not reveal sex differences concerning this association. The table also demonstrates that the predictors impacting SRH after 2 years, also impacted the change in SRH during the two-year time span, making causal inference more trustworthy.

**Table 2:** Temporal causal and residual change analyses of predictors in 2011 with self-rated health in 2013 as outcome. ORs with 95% CIs not including 1 marked with bold

Variables	Temporal causal, OR^a^	95% CI	Explained variance, Nagelkerke	Residual change, OR^b^	95% CI	Explained variance, Nagelkerke
***Adjusting variables***						
Sex, girls, ref	1			1		
Boys	**2.0**	1.6, 2.5		**1.8**	1.5, 2.3	
Age group, 15 yrs., ref	1			1		
13 yrs	**1.4**	1.1, 1.7		1.2	1.0, 1.6	
Family affluence	**1.4**	1.2, 1.7	0.06	1.2	1.0, 1.4	
Self-rated health, 2011						
Very good				1		
Good				**0.3**	0.2, 0.4	
Not so good/ bad				**0.1**	0.1, 0.1	0.18
***Predictor variables***						
BMI 2011	**0.9**	0.9, 0.9	0.11	**0.9**	0.9, 1.0	0.21
Self-esteem 2011	**2.5**	2.0, 3.2	0.11	**1.7**	1.3, 2.2	0.20
***Want to change something about body***						
Yes	1			1		
No	**1.8**	1.4, 2.3	0.08	**1.4**	1.1, 1.8	0.19
***Evaluate my body***						
It is OK or I don’t think about it	**2.4**	1.8, 3.0		**1.6**	1.2, 2.1	
It is too thin	**2.9**	2.0, 4.4		**2.2**	1.5, 3.3	
It is too fat	1		0.10	1		0.19
***Dieting***						
No, my weight is fine	**2.3**	1.7, 3.1		**1.8**	1.3, 2.4	
No, I need to gain weight	**3.4**	2.0, 5.6		**3.0**	1.8, 5.1	
No, but I need to lose weight	1.0	0.7, 1.6		1.1	0.7, 5.1	
Yes. I am dieting	1		0.10	1		0.20

^a^The adjusting variables are presented from an analysis with all the adjusting variables in the model, and thereafter each of the predictors are entered one by one in multivariate models with the corresponding explained variance.

^b^Residual change analyses, adjusting for self-rated health in 2011, are performed with each of the predictors entered with the corresponding explained variance

In Tables 3 and 4 we demonstrated that among boys SRH, BMI and the body and weight concern variables measured in 2011 impacted SE in 2013 in the same manner as they impacted SRH. We revealed standardised beta coefficients varying from − 0.09 (CI = − 0.17,-0.01) to − 0.19 (CI = − 0.27, − 0.11) among boys and from − 0.19 (CI = − 0.27, − 0.11) to − 0.29 (CI = −-0.37, − 0.21) among girls. We also noted with this outcome that it was the concern of being too fat and needing to lose weight or engaging in dieting that impacted SE, not the concern of being too thin or needing to gain weight. We confirmed the importance of these predictors with residual change analyses only among girls, inferring that the body and weight concern predictors in 2011 impacted the change in SE during the 2 years’ observation. Among boys SRH had a significant impact on the change in SE, not seen among girls.

**Table 3:** Temporal causal and residual change analyses of predictors in 2011 with self-esteem in 2013 as outcome ***among boys***. Standardised beta coefficients (b) with 95% CIs not including 0 marked with bold

Variables	Temporal causal, Standardised b^a^	95% CI	Explained variance	Residual change, Standardised b^b^	95% CI	Explained variance
***Adjusting variables***						
Age group, 15 yrs	−0.02	− 0.11, 0.06		0.01	−0.06, 0.13	
13 yrs., ref	0			0		
Family affluence	0.07	− 0.01, 0.11	0.00	− 0.01	− 0.06, 0.05	
Self-esteem, 2011				**0.43**	0.35, 0.51	0.18
***Predictor variables***						
Self-rated health 2011						
Very good	**0.33**	0.19, 0.49		0.09	−0.06, 0.26	
Good	**0.30**	0.15, 0.44		**0.14**	0.00, 0.28	
Not so good/ bad, ref	0		0.03	0		0.18
BMI 2011	**−0.10**	−0.20, − 0.01	0.01	− 0.07	−0.15, 0.01	0.18
***Want to change something about body***						
Yes	**− 0.19**	−0.27, − 0.11		−0.07	− 0.15, 0.01	
No, ref	0		0.04	0		0.18
***Evaluate my body***						
It is OK or I don’t think about it, ref	0			0		
It is too thin	−0.02	− 0.10, 0.06		0.00	−0.11, 0.11	
It is too fat	**−0.11**	− 0.19, − 0.02	0.01	−0.02	− 0.09, 0.05	0.18
***Dieting***						
No, my weight is fine, ref	0			0		
No, I need to gain weight	−0.02	− 0.10, 0.06		0.01	−0.06, 0.08	
No, but I need to lose weight	**−0.14**	− 0.22, − 0.06		− 0.05	− 0.13, 0.03	
Yes. I am dieting	**− 0.09**	− 0.17, − 0.01	0.02	− 0.01	− 0.09, 0.07	0.18

^a^The adjusting variables are presented from an analysis with all the adjusting variables in the model, and thereafter each of the predictors are entered one by one in multivariate models with the corresponding explained variance.

^b^Residual change analyses, adjusting for self-esteem in 2011, are performed with each of the predictors entered with the corresponding explained variance

**Table 4:** Temporal causal and residual change analyses of predictors in 2011 with self-esteem in 2013 as outcome ***among girls***. Standardised beta coefficients (b) with 95% CIs not including 0 marked with bold

Variables	Temporal causal, Standardised b^a^	95% CI	Explained variance	Residual change, Standardised b^b^	95% CI	Explained variance
***Adjusting variables***						
Age group, 15 yrs	**−0.20**	− 0.27, − 0.12		**− 0.10**	−0.17, 0.03	
13 yrs., ref	0			0		
Family affluence	**0.12**	0.04, 0.20	0.05	0.02	−0.05, 0.09	
Self-esteem, 2011				**0.45**	0.37, 0.53	0.23
***Predictor variables***						
Self-rated health 2011						
Very good	**0.28**	0.15, 0.41		0.03	−0.10, 0.16	
Good	**0.16**	0.03, 0.28		−0.01	− 0.16, 0.13	
Not so good/ bad, ref	0		0.08	0		0.23
BMI 2011	−0.07	− 0.16, 0.02	0.05	− 0.01	− 0.05, 0.03	0.23
***Want to change something about body***						
Yes	**−0.29**	−0.37, − 0.21		**− 0.18**	− 0.26, − 0.10	
No, ref	0		0.12	0		0.26
***Evaluate my body***						
It is OK or I don’t think about it, ref	0			0		
It is too thin	0.06	−0.02, 0.14		0.07	− 0.01, 0.14	
It is too fat	**−0.25**	−0.33, − 0.17	0.12	**−0.13**	− 0.21, − 0.05	0.25
***Dieting***						
No, my weight is fine, ref	0			0		
No, I need to gain weight	0.01	−0.06, 0.08		0.02	−0.05, 0.09	
No, but I need to lose weight	**−0.19**	−0.27, − 0.11		**−0.11**	− 0.19, − 0.03	
Yes. I am dieting	**− 0.22**	− 0.30, − 0.14	0.11	**− 0.12**	− 0.20, − 0.04	0.25

^a^The adjusting variables are presented from an analysis with all the adjusting variables in the model, and thereafter each of the predictors are entered one by one in multivariate models with the corresponding explained variance.

^b^Residual change analyses, adjusting for self-esteem in 2011, are performed with each of the predictors entered with the corresponding explained variance.

Table 5 reveals the predictive associations with BMI in 2013 as outcome. It is worth noting that positive resilience factors like SE and SRH were associated with a leaner body after 2 years. Standardised beta coefficients for SE were − 0.11 (CI = − 0.17,-0.04) and for the two levels of SRH -0.11 (CI = − 0.20,-0.06) and − 0.25 (CI = − 0.35,-0.15) respectively. Positive SRH even predicted BMI reductions during the 2 years (b = − 0.14, CI = − 0.25,-0.04), compared with adolescents with impaired SRH. We also demonstrated that the body and weight concern factors impacted BMI in a paradoxical manner. The intention of gaining weight led to a leaner body (b = − 0.12, CI = − 0.18,-0.06), and the intention of losing weight led to a heavier body during the 2 years (b = 0.15, CI = 0.09,0.21). Similarly, engaging in dieting led to weight gain during the 2 years (b = 0.20, CI = 0.14,0.26). The explained variance gain in the residual change analyses testifies that these associations were important.

**Table 5:** Temporal causal and residual change analyses of predictors in 2011 with BMI (LogBMI) as outcome. Standardised beta coefficients (b) with 95% CIs not including 0 marked with bold

Variables	Temporal causal, Standardised b^a^	95% CI	Explained variance	Residual change, Standardised b^b^	95% CI	Explained varriance
***Adjusting variables***						
Sex, girls	0.03	−0.03, 0.08		0.03	−0.03, 0.08	
Boys, ref	0			0		
Age group, 15 yrs., ref	**0.30**	0.25, 0.36		**0.22**	0.16, 0.28	
13 yrs	0			0		
Family affluence	**−0.07**	−0.13, −0.01	0.10	−0.05	− 0.11, 0.03	
BMI 2011				**0.41**	0.32, 0.46	0.25
***Predictor variables***						
Self-esteem 2011	**−0.11**	−0.17, − 0.04	0.10	− 0.06	−0.13, 0.00	0.26
Self-rated health, 2011						
Very good	**−0.25**	−0.35, − 0.15		**− 0.14**	−0.25, − 0.04	
Good	**−0.11**	− 0.20, − 0.06		−0.07	−0.18, 0.03	
Not so good/ bad, ref	0		0.12	0		0.26
***Want to change something about body***						
Yes	**0.16**	0.10, 0.22		**0.09**	0.04, 0.16	
No, ref	0		0.12	0		0.26
***Evaluate my body***						
It is OK or I don’t think about it, ref	0			0		
It is too thin	**−0.20**	−0.26, −0.15		**−0.15**	−0.21, −0.09	
It is too fat	**0.32**	0.26, 0.38	0.26	**0.22**	0.16, 0.28	0.32
***Dieting***						
No, my weight is fine	0			0		
No, I need to gain weight	**−0.16**	−0.21, −0.10		**−0.12**	−0.18, − 0.06	
No, but I need to lose weight	**0.23**	0.17, 0.29		**0.15**	0.09, 0.21	
Yes. I am dieting	**0.28**	0.23, 0.34	0.24	**0.20**	0.14, 0.26	0.32

^a^The adjusting variables are presented from an analysis with all the adjusting variables in the model, and thereafter each of the predictors are entered one by one in multivariate models with the corresponding explained variance.

^b^Residual change analyses, adjusting for self-esteem in 2011, are performed with each of the predictors entered with the corresponding explained variance.

## Discussion

### Objectives and main findings

We set out to examine how body weight and body shape concerns predicted SRH and SE in a general adolescent population after 2 years; how the reciprocal associations between these outcomes were during the 2 years; and how predictors influenced *change* in the outcomes during the two-year time span.

The study revealed that body and weight concerns have unfavourable effects on subjective health and self-esteem, and that SRH and SE have mutually favourable effects on each other. Increased body mass had unfavourable effects on SRH, but these were less so for SE. The impact of body and weight concerns on SE was particularly strong among girls. In addition, we revealed that the intention of becoming thinner or fatter had strong paradoxical effects on body mass during the 2 years of the survey: the intention of getting thinner and engaging in dieting were associated with a BMI increase, and the intention of getting fatter was associated with a BMI decrease. Positive SRH and SE were both associated with a leaner body after 2 years, and SRH was also associated with a beneficial BMI change during the 2 years’ observation.

The predictive associations that we found between BMI and impaired SRH and SE are not always confirmed in the literature. In a cross-sectional study in three European countries, among somewhat older adolescents, these associations were not supported [23]; whereas adults participating in a lifestyle intervention study in Spain exhibited similar and reciprocal associations [24]. Also, in an intervention study with mostly overweight and obese adults, two of the authors of the present study (EM and GBS) revealed complex and reciprocal relations, similar to those found in the present study [25].

### What is already known on this topic

SE and SRH were reciprocally related in the present study. Our findings are in line with previous research, that high levels of SRH at inclusion significantly predict improvements in self-conceptual measures, e.g. SE and body shape concern. Earlier studies often had a cross-sectional design [26, 27], however, and our findings add support for the mutual and reciprocal link over time between SRH and SE.

We also revealed that both SRH and SE were associated with a leaner body after the 2 years. The causal link was supported as high SRH was associated with less weight gain during the 2 years. In some studies, impaired SE served as a significant predictor for short-term, but not for long-term, weight loss [28]. In line with the present findings, body satisfaction predicted a leaner body, whereas self-evaluative discontent with the body was related to weight gain [29]. In addition, weight labelling from others predicted weight gain in early adolescence [6]. In a review aimed at identifying pre-treatment factors for successful weight loss, positive and autononomous motivation were associated with success, whereas other factors, including body image, SE, and weight-specific quality of life, exhibited an inconsistent influence on later BMI [30].

### What this study adds

Overall, it appears that dissatisfaction and body distress may hinder attempts to lose weight, although multiple factors might confound this association [31]. There is increasing concern, however, that a focus on weight is not only ineffective at producing thinner and healthier bodies, but may also have unintended consequences. It may contribute to food and body preoccupation, repeated cycles of weight loss and regain, and distraction from more sustainable health engagement. Reduced SE, eating disorders, and weight stigmatisation and discrimination could follow in the wake of a preoccupation with weight [2].

The results of the present study add evidence that these concerns are relevant. The various measures that we have used to describe body dissatisfaction are all associated with impairments in SRH and SE, both in temporal causal analyses and in residual change analyses. In addition, earlier research showed that body shape and weight concerns among adolescents have long-term health consequences, with increased infectious and other somatic morbidity in early adulthood [32]. We have also documented a paradoxical effect of body dissatisfaction: that being eager to become thinner makes you fatter, and being eager to get fatter makes you thinner, compared with peers who are content with their bodies. Certainly, there are contesting explanations for these seemingly self-contradictory results. Genetic and other factors impacting weight regulation, working beyond and independently of human aspiration, are candidates for an alternative explanation [33].

Independent of such explanations, we are exposed to a dilemma: carrying excess weight and obesity are associated with ill health [1], but the clinical and public health efforts aimed at weight reduction may simultaneously represent a double-edged sword with unintended health impairments and paradoxical effects as results. Community- and school-based interventions are promising and may protect against stigmatising effects and body dissatisfaction [34, 35]. Non-diet interventions based on intuitive and mindful eating have led to weight reduction in studies with non-intervention controls [36], but the most important effects were that they promoted self-esteem, respect for body size diversity, and mitigated eating disorders [2]. Some researchers, therefore, are calling for behaviour change approaches that improve psychological well-being in schools and in the general population [12, 37], e.g. SE, body satisfaction, SRH, and quality of life.

Although the results of this observational study cannot inform clinical work directly, we maintain that they lend support to health promotive efforts using weight neutral approaches, which also aim at improved subjective health and wellbeing in the clinical encounter [2, 36]. In a clinical trial performed by two of the authors of the present study (EM and GBS), BMI reduction was predicted by self-efficacy for physical activity and autonomous motivation for change. Positive self-concepts, e.g. self-efficacy, improved both BMI and body attitude simultaneously during follow-up [25].

### Limitations and strengths of this study

The weaknesses of the study include the large proportion of students lost to follow-up, and possibly also the context of the study being set in mostly rural districts of western Norway. The large drop-out rate due to insufficient coding, especially in the youngest age group, represented a threat to the external validity of the study. Selective drop-out of those with better subjective health and fewer concerns with their own body may reduce the generalisability of the study. The results are from a narrow age group, and extrapolating the results to other age groups should be approached with caution. The drop-outs were random and not associated with participant characteristics. It is, therefore, unlikely that the predictive associations demonstrated in the study are invalid. Reliance on self-reports and an identical questionnaire at both time-points introduce the possibility of common method variance.

The strengths of the study are its longitudinal design and the evaluation of several predictors and outcomes. We examined both subjective health impairment, body mass and self-conceptual problems. Both mediation and moderation were examined as we adjusted for SES and reported sex stratified analyses. We performed both temporal causal and residual change analyses. The study also adjusted for possible confounders, the most important of which was the self-rated SES that is linked with both body mass and subjective health.

The former county of Sogn og Fjordane is mainly a rural district, although urban areas exist. This setting may represent a threat to the external validity of the study. In several studies, however, two of the authors (EM and HJB) have demonstrated that adolescent health and health behaviour problems are similar to national and even international findings [38]. We therefore maintain that the external validity of our findings seems safeguarded.

## Conclusion

In conclusion this study confirms that BMI, SE SRH, body shape and body weight concerns were reciprocally associated with complex inter-relations. Body shape and weight concerns predicted impaired SRH and SE, whereas positive SRH and SE predicted a leaner body. Health promotion strategies, therefore, built on positive self-concepts and body acceptance should be increasingly emphasised both in clinical and in public health practice.

## Key points

- Obesity in childhood and adolescence is associated with later health loss
- The causality of this association is complex and insufficiently understood
- In an early adolescent general population self-rated health and self-esteem predicted a leaner body
- Body shape and weight concerns predicted deteriorating self-rated health and self-esteem
- Weight focus may have unwanted side effects
- Health promotion should build on positive self-concepts and body acceptance

## Supplementary Information

**Additional file 1.**

## Data Availability

The raw data used in this study can be obtained from the Norwegian Center for Research data https://www.nsd.no/en after reasonable request to the corresponding author.

## Abbreviations

BMI: Body Mass Index
SE: Self-esteem
SRH: Self-rated health
SES: Socio-economic status
HBSC: Health in School-Aged Children
