# Supplementary material for: How body concerns, body mass, self-rated health and self-esteem are mutually impacted in early adolescence: a longitudinal cohort study
Source: BMC Public Health. 2021 Mar 12;21:496. doi: 10.1186/s12889-021-10553-x (PMC7953559; doi:10.1186/s12889-021-10553-x)
Supplement: Supplementary file 1 — Additional file 1. [file 12889_2021_10553_MOESM1_ESM.docx]

**Appendix 1. Questionnaire used in the survey**

|  | **School Health Service in THE MUNICIPALITY** |
| --- | --- |

HEALTH HABITS, ENVIRONMENT AND LIFESTYLE AMONG STUDENTS IN 2011.

Survey among pupils in in 6th, 8th and 10th class at schools autumn 2011.

|  |  |
| --- | --- |

Serial number:

Parents has approved: Yes  No

I will not participate: (the form will be discarded)

1. **Are you a boy or a girl? (Put x in the box that fits)**

Boy  girl

1. **What class do you belong to?**

6th class  8th  10th

**5. How heavy are you undressed?**  My weight is kilos

**6. What is your height without shoes?**  I'm cm

**11. How wealthy is your family?**

Very wealthy  Wealthy  Average

Not particularly wealthy  We are not wealthy

**24. How do you estimate your current health?**

It is::

Very good

Good

Not so good

Bad

**-----------------------------------------------------------------------------------**

**Now more questions about how you think about yourself.**

**-----------------------------------------------------------------------------------**

**25. Is there something about your body you want to change?**

Yes

No

**26.What do you think about your body?**

It is:

Too thin

A little too thin

About as it should be

A little too fat

Too fat

I don't think about it.

**27.Are you trying to slim down?**

No, my weight is fit

No, but I think that I need to slim

No, because I need to put on weight

Yes

**44. What kind of opinion do you have of yourself?** Check the box of quare phrases below that is most suitable for you.

To what degree do you agree/ disagree

Very agree Agree Disagree Very disagree

a) Over all, I am satisfied with myself

b) I feel at times that I don't manage

c) I feel that I have many good qualities

d) I am able to perform things just as

good as others

e) I feel that I don't have much to be

proud of

f) I feel useless at times

g) I feel as a person who is

equal to others

h) I wish I had more self-respect

i)Over all, I consider myself to be

rather missy.

j) I have a positive perception of

myself
